# Male Down syndrome Ts65Dn mice have impaired bone regeneration

**DOI:** 10.1016/j.bone.2024.117374

**Published:** 2024-12-13

**Authors:** Kirby M. Sherman, Catrina J. Silveira, Mingquan Yan, Ling Yu, Abigail Leon, Kasey Klages, Lauren G. White, Hannah M. Smith, Sarah M. Wolff, Alyssa Falck, Ken Muneoka, Regina Brunauer, Dana Gaddy, Larry J. Suva, Lindsay A. Dawson

**Affiliations:** a Department of Veterinary Physiology and Pharmacology, College of Veterinary Medicine and Biomedical Sciences, Texas A&M University, College Station, TX 77843, United States of America; b LBG Ludwig Boltzmann Institute for Traumatology, The Research Center in Cooperation with AUVA, 1200 Vienna, Austria; c Austrian Cluster for Tissue Regeneration, 1200 Vienna, Austria; d Department of Veterinary Integrative Biosciences, College of Veterinary Medicine and Biomedical Sciences, Texas A&M University, College Station, TX 77843, United States of America

**Keywords:** Digit, Down syndrome, Bone, Ts65Dn, Regeneration, P3, Dp16

## Abstract

Trisomy of human chromosome 21 (Ts21) individuals present with a spectrum of low bone mineral density (BMD) that predisposes this vulnerable group to skeletal injuries. To determine the bone regenerative capacity of Down syndrome (DS) mice, male and female Dp16 and Ts65Dn DS mice underwent amputation of the digit tip (the terminal phalanx (P3)). This is a well-established mammalian model of bone regeneration that restores the amputated skeletal segment and all associated soft tissues. P3 amputation was performed in 8-week-old male and female DS mice and WT controls and followed by *in vivo* μCT, histology and immunofluorescence. Following P3 amputation, the bone degradation phase was attenuated in both Dp16 and Ts65Dn males. In Dp16 males, P3 regeneration was delayed but complete by 63 days post amputation (DPA); however, male Ts65Dn exhibited attenuated regeneration by 63 DPA. In both Dp16 and Ts65Dn female DS mice, P3 regenerates were indistinguishable from WT by 42 DPA. In Ts65Dn males, osteoclasts and eroded bone surface were significantly reduced, and osteoblast number significantly decreased in the regenerating digit. In Ts65Dn females, no significant differences were observed in any osteoclast or osteoblast parameter. Like Ts21 individuals and DS mice with sex differences in bone mass, these data expand the characteristic sexually dimorphism to include bone resorption and regeneration in response to skeletal injury in Ts65Dn mice. These observations suggest that sex differences contribute to the poor bone healing of DS and compound the increased risk of bone injury in the Ts21 population.

## 1. Introduction

Down syndrome (DS), the result of triplication of human chromosome 21 (Hsa21) is the most viable human trisomy, occurring in around 1:700 live births [1]. Trisomy 21 (Ts21) impacts multiple organ systems during development and causes a complex disease spectrum in Ts21, including hallmark skeletal deficits, such as short stature and craniofacial alterations. The increasing life expectancy of individuals with Ts21 [2,3] has fostered a growing awareness of mid- and late-life conditions, such as low bone mineral density (BMD). Collectively, Ts21 individuals present with a wide spectrum of low BMD that predisposes this vulnerable group to skeletal injuries [4–9] with male Ts21 individuals displaying an earlier onset and greater propensity for lower BMD than Ts21 females [4,10,11].

We have previously shown that fracture healing is significantly impaired in DS mice which suggests an inherent problem with the ability of bone to heal and has been postulated to be inherent in Ts21 individuals [4,5,11,12]. In line with this idea, a recent case report highlighted an atrophic non-union in a female Ts21 infant [13], which is the first published case of impaired bone healing in Ts21. Moreover, the first retrospective cohort study of adults ≥18 years of age with Ts21 demonstrated a significant positive association between individuals with Ts21 and subsequent fractures [14]. These examples underscore the utility of DS mouse models to explore low bone mass, and impaired fracture healing may predict clinical Ts21 bone healing scenarios. Our previous fracture healing studies in DS mice suggested that the failure to form the soft callus is an early defect that impairs the process of endochondral bone formation, formation of the hard callus and eventual fracture repair [5]. This observation led us to utilize a well characterized mammalian model of bone regeneration, the regeneration of the amputated digit tip, the terminal phalanx (P3) in mice, to investigate intramembranous bone regeneration independent of intermediate endochondral action, as no cartilage callus formation is required to fully regenerate the entire digit tip [15–17].

The mouse digit tip has extensive amputation-level-dependent regenerative capabilities reminiscent of those observed in amputated digit tips of young and adult humans [18–20]. The P3 regenerative response involves the formation of a blastema and thus the mouse digit tip is a mammalian model of epimorphic regeneration [15,21–23]. The P3 blastema is a transient structure comprised of proliferating cells that give rise to the regenerated structures, including the P3 bone [24–26]. P3 amputation initiates robust bone regeneration within 42 days [15,27] with bone regeneration characterized by an initial phase of osteoclast-mediated bone degradation (osteolysis) with simultaneous blastema formation. This is followed by wound closure and subsequent blastemal-derived osteoblast differentiation and robust direct bone regeneration *via* intramembranous ossification. This leads to the full recapitulation of the entire P3 digit along with the surrounding soft tissues. Thus, in this model, targeted osteoclast and osteoblast activities can be spatially and temporally investigated *in vivo* over the course of bone regeneration [15,17,24,26,28,29].

There are numerous mouse models of Down syndrome [2,12,30], with which we and others have reported the basal bone phenotypes [5,31–34]. However, few publications have investigated fracture healing [5] with even less focus on the bone regenerative and repair capacity of the DS mouse skeleton. Of the many murine DS models available [2], the Dp16(16)1Yey mice (Dp16) and Ts65Dn DS mouse strains are the most well-investigated and well-established [35]. In both models, genes found on *Hsa*21 are spread across three mouse chromosomes (*Mmu*) *Mmu*10, 16 and 17 [2]. In Dp16 DS mice, the entire *Hsa*21 syntenic region of *Mmu*16 is duplicated, resulting in trisomic expression of this region [36,37]. Ts65Dn DS mice are characterized by segmental trisomy for a region of *Mmu16* that contains approximately 75 % of *Hsa21*-homologous genes [35]. Both Ts65Dn and Dp16 DS mice exhibit the characteristic behavioral, reproductive, craniofacial, and cardiac phenotype of people with Ts21, including the well-described sexual dimorphism in bone mass [4,5,11,38]. Additionally, we and others have shown that just as in people with Ts21, the Dp16 and Ts65Dn mice have low bone volume and significantly decreased bone formation rate (BFR) [5,31–34,39] compared to wildtype controls. Following P3 amputation in both the Dp16 and Ts65Dn DS mouse models, we have discovered in the current study that Ts65Dn (but not Dp16) mice display sexually dimorphic responses across the P3 regeneration cascade. Specifically, females of both DS strains regenerate P3 normally with only Ts65Dn males displaying significantly impaired P3 regeneration. These data amplify our interest in the spectrum of bone phenotypes and increased risk of bone injury that is inherent in the Ts21 population.

## 2. Materials and methods

### 2.1. Animals and P3 amputation

Trisomic female B6EiC3Sn *a*/*A*-Ts(17^16^)65Dn/J (Ts65Dn; strain #001924) mice and Wildtype (WT) male B6EiC3SnF1/J (strain #001875) mice were purchased from The Jackson Laboratory (Bar Harbor, ME) and bred at the Texas A&M Institute for Genomic Medicine (TIGM). Hemizygous male B6.129S7-Dp(16Lipi-Zbtb21)1Yey/J (Dp16; strain #013530) mice and WT female C57BL/6 J (strain #000664) mice were purchased from The Jackson Laboratory and bred at TIGM. All mice purchased from The Jackson Laboratory had their genotype confirmed upon delivery, and all resulting mice bred in-house were genotyped using established and published protocols [5,31,32]. At TIGM, all mice were maintained on a 12-h light/dark cycle and had *ad libitum* access to water and standard rodent chow. All baseline P3 analysis and P3 amputation experiments were performed in 8-week-old male and female mice. P3 amputation has been previous described in detail [15,24,26,40]. Briefly, mice were anesthetized using isoflurane, with an initial dose of 3 % followed by a 2 % maintenance dose over the course of the surgery. P3 amputation was carried out on both hind limb digits 2 and 4, for up to a total of 4 digits per mouse, with digit 3 serving as an uninjured internal control [15]. All mice were treated similarly, regardless of genotype. Digits were imaged by *in vivo* microcomputed tomography (μCT) prior to amputation and up to 63 days post amputation (DPA) to assess bony anatomy. Digit staging by μCT was performed as described [17]. Unamputated, as well as 7 DPA, 10 DPA, and 14 DPA digits were harvested and processed for histology and evaluation. All animal use followed the standard operating procedures of the Texas A&M University IACUC under a Texas A&M University IACUC approved AUP.

### 2.2. Digit processing, histology, immunohistochemistry, and image analysis

Digit processing has been previously described [15,17,27]. Briefly, digits were fixed in 10 % Neutral Buffered Formalin for 24–96 h at room temperature. Following fixation, digits were decalcified using Decalcifier 1 (Surgipath, Leica Biosystems, Richmond, IL) for 20–24 h at room temperature. Digits were processed and embedded in paraffin and sectioned at 4 μm. For all histology and immunohistochemistry, slides were incubated at 65 °C for 45 min followed by 37 °C for no <15 min prior to deparaffinization. For immunostaining, antigen heat retrieval (pH 6 citrate buffer, Invitrogen, Carlsbad, CA) was carried out by incubating slides in citrate buffer overnight at 65 °C to maintain tissue morphology. Slides were then incubated using Protein Block Solution (DAKO) for 45 min to 1 h at room temperature. Slides were incubated in primary antibodies overnight at 4 °C. The following primary antibodies were used: monoclonal mouse anti-Proliferating Cell Nuclear Antigen (PCNA) (Abcam; ab29, 0.5 μg/ml); rabbit anti-Cathepsin K (CTSK) (Abcam; ab19027, 2 μg/ml); and rabbit anti-Sp7/Osterix (OSX) monoclonal antibody (Abcam; ab22552, 0.125 μg/ml). Slides were incubated for 45 min at room temperature using the following secondary antibodies: Alexa Fluor goat anti-rabbit 488 IgG secondary antibody (Invitrogen, A11008, 1:500 dilution) and Alexa Fluor goat anti-mouse 647 IgG secondary antibody (Invitrogen, A21235, 1:500 dilution). Samples were counterstained with DAPI for 5 min. Samples were imaged using 1) the Olympus BX61 fluorescence deconvolution microscope using Slide-book software (Intelligent Imaging Innovation Inc., Denver, CO), 2) the Olympus VS120 microscope with a Hamamatsu ORCA-Flash 4.0 camera using VS-ASW Fl2.8 software (Olympus), followed by image processing using Fiji [41] and the BIOP VSI Reader [42] using a standardized workflow [29], and 3) the Olympus BX60 microscope and Olympus DP72 camera, with image processing using the DP2-BSW software (Olympus America Inc., Center Valley, PA). All immunohistochemical and histological assessments were carried out blinded to genotype. Quantification of osteoclasts, osteoblasts, and proliferating cells was performed in a medial section from each digit by assessing positive immunostaining from the P3 joint to the distal tip and included all tissue bounded by the dorsal nail epithelium and the ventral epidermis as described [17,29]. Osteoclast quantification was carried out by identifying CTSK^+^ cells containing three or more nuclei and localized to the P3 bone surface, followed by normalizing to total bone perimeter. Mallory Trichrome stained serial sections were analyzed to obtain total bone perimeter and eroded bone surface. DAPI, PCNA, and OSX^+^ nuclei were counted manually, and OSX and PCNA were normalized to the total number of DAPI cells.

### 2.3. Microcomputed tomography (μCT) scanning

*In vivo* microcomputed tomography (μCT) scanning was performed prior to P3 amputation and on regenerating digits over the course of regeneration as previously described in detail [15,17,26,29,43,44] using the vivaCT 40 (SCANCO Medical, Wayne, Pennsylvania). The P3 digit region was scanned at a voxel size of 10.5 μm, at 55 kVp, 145 μA, 300 msec integration time, with 1000 projections per 180° using continuous rotation, in accordance with standard ASBMR practices [45]. The ROI covered approximately 2 mm, and the μCT dose index (CTDI) was 960 mGy per scan [17]. Image generation and analysis of bone volume and length was performed using the BoneJ Plugin for Image J (Fiji) [46] as previously described [15]. Using the CT scan, digit bone length was calculated from the central base of the P3 joint extending to the distal-most bone (as shown by the dashed red line in Fig. 2A) or bone island as described [15]. Pseudocoloring of staged digits was performed in PowerPoint.

### 2.4. Statistics

Sample size throughout the study consisted of 8–48 digits. Based on our previous investigation of P3 regeneration in mice [15–17,29,43,44,47,48], a power analysis prior to the start of the study indicated n = 6 digits per group would provide sufficient power to detect a significant difference for all endpoints. All statistical tests were carried out using GraphPad PRISM, version 9.5.1 (2023), (GraphPad Software, La Jolla, CA). For changes in bone length and volume over the course of regeneration, significance between groups was measured by multiple *t-*tests with Holm-Sidak correction at each time point. Immunohistochemical data was analyzed using a Two-way ANOVA with Tukey’s multiple comparisons test. All data are expressed using violin plots showing all data points, medians, and 1st and 3rd quartiles for each group. Wound closure was compared between groups by summing closed *versus* open wounds, respectively, across all analyzed time points using the Fisher’s exact test. Wound closure P values indicate significance across all assessed time points.

## 3. Results

### 3.1. Sexually dimorphic bone regeneration responses in Dp16 and Ts65Dn DS mice

**Fig. 1.**
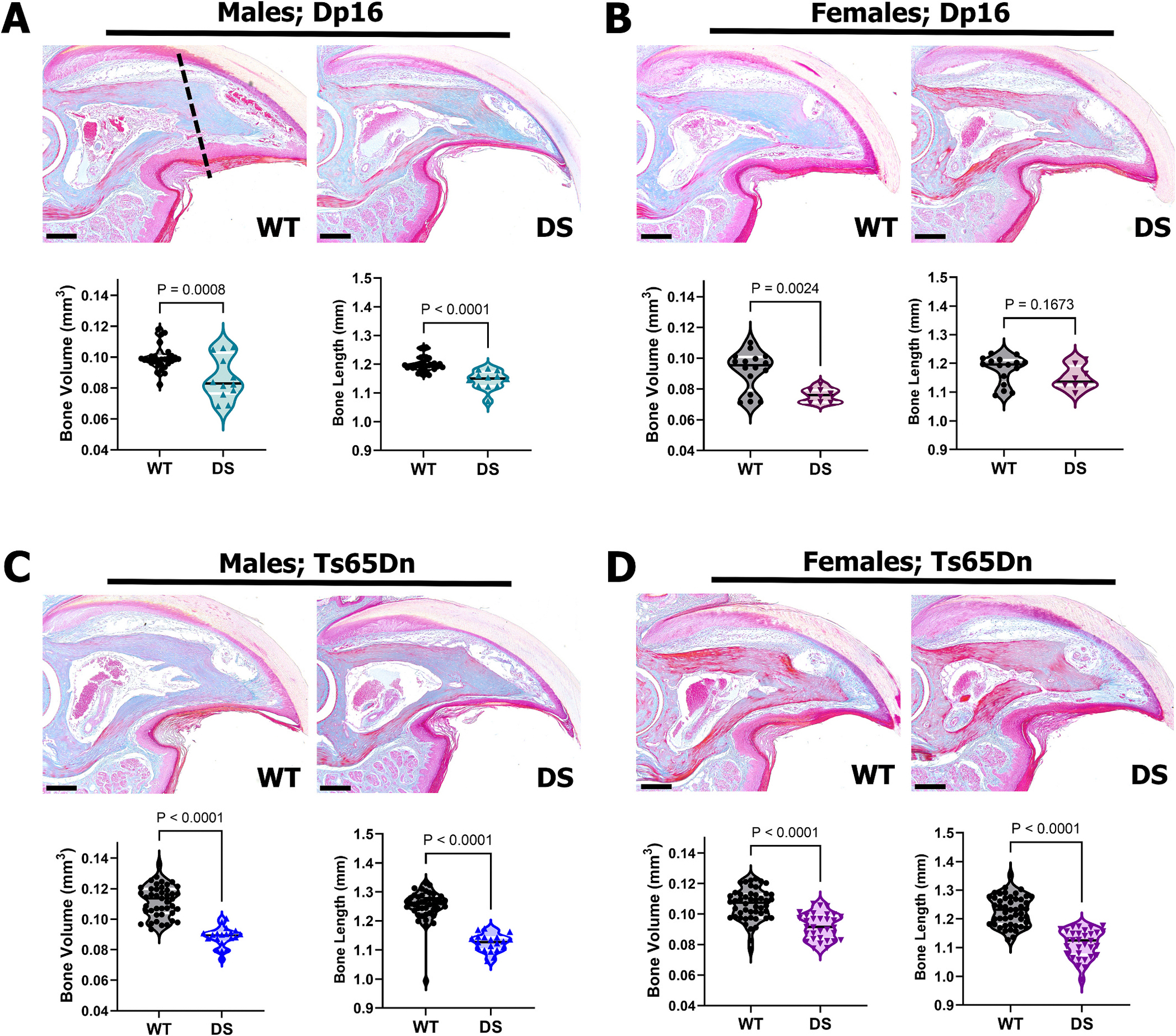
Altered baseline P3 bone volume and length in male and female 8-week-old Dp16 and Ts65Dn mice. Distal is to the right, dorsal is to the top. Violin plots showing individual data points, medians and 1st and 3rd quartiles resulting from two-way ANOVA, with Tukey’s multiple comparisons test. (A) Mallory staining of representative WT and Dp16 DS male unamputated P3 digits; dashed line denotes standardized amputation plane. Dp16 DS males exhibited significantly decreased bone volume and length compared to WT males. (B) Mallory staining of representative WT and Dp16 DS female unamputated P3 digits. Dp16 DS females have decreased P3 bone volume compared to WT controls yet show no differences in bone length. (C) Mallory staining of representative WT and Ts65Dn DS male unamputated P3 digits. Ts65Dn DS males have significantly decreased bone volume and length compared to WT males. (D) Mallory staining of representative WT and Ts65Dn DS female unamputated P3 digits. Ts65Dn DS females have significantly decreased bone volume and length compared to WT females. Scale bars = 200 μm.

Our previous studies demonstrated that 3–6-month-old male Dp16 DS mice exhibit low bone mass and volume compared to euploid wild-type controls, whereas age-matched female Dp16 DS mice are comparable to euploid controls [5]. Moreover, previous studies have demonstrated similar sexually dimorphic skeletal deficits in male and female Ts65Dn mice [38,50]. To determine whether skeletal differences observed in long bones was observed in the terminal phalanx (P3) bone at-baseline, μCT scanning and histologic evaluation of P3 digits of young 8-week-old male and female Dp16 and Ts65Dn DS mice and euploid WT controls was performed (Fig. 1). P3 is a triangularly shaped bone encased by the nail organ on the dorsal and lateral surfaces, with the epidermis on the ventral surface. Histological analysis did not identify overt P3 skeletal, marrow, nail, or soft tissue alterations between DS and WT mice (Fig. 1A–D). However, μCT analysis of unamputated P3 digits demonstrated significantly decreased P3 bone volume in both male (n = 12 digits) and female Dp16 (n = 8 digits) mice compared to WT males (n = 28 digits) and females (n = 16 digits) (Fig. 1A, B), consistent with our findings in the tibia of 3–6 month old Ts65Dn [32] and Dp16 DS mice [5]. The 8-week-old Dp16 males also exhibited significantly decreased P3 bone length compared to WT males. In contrast, Dp16 DS and WT females were not significantly different in P3 bone length (Fig. 1A, B). Similarly, in Ts65Dn DS mice, both males (n = 20 digits) and females (n = 32 digits) had significantly reduced P3 bone and length compared to WT males (n = 43 digits) and females (n = 48 digits) (Fig. 1C, D) as we and others have shown for the bones of the axial and appendicular skeleton in male and female Ts65Dn DS mice [31–33,38,39,50,51].

**Fig. 2.**
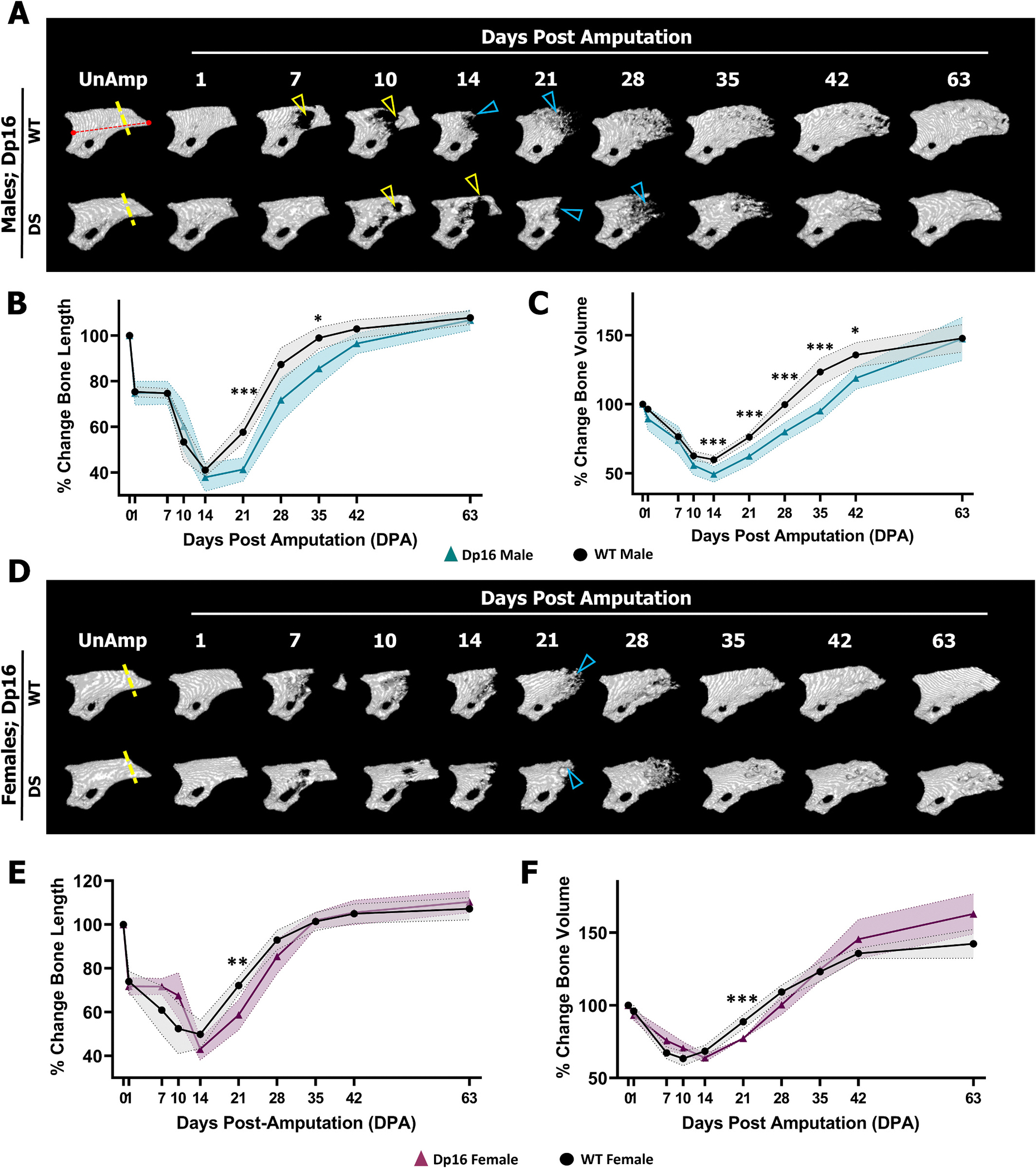
Transiently attenuated P3 bone regeneration in male and female Dp16 mice. Distal is to the right, dorsal is to the top. (A) Representative μCT renderings of a single WT male (top) P3 digit and a single Dp16 DS male (bottom) P3 digit prior to amputation (yellow dashed line) and over the duration of P3 regeneration. The red dashed line denotes the region used for measuring bone length. The P3 degradation response (yellow arrowheads) expels the necrotic bone fragment and is followed by blastema osteogenic differentiation and mineralization (blue arrowheads) to regenerate the P3 bone. (B) Dp16 DS males exhibit transiently attenuated gains in bone length at 21 (***P = 0.000469) and 35 (*P = 0.015568) days post amputation (DPA) that are resolved by 42 DPA. (C) P3 regeneration in Dp16 DS males is characterized by enhanced bone degradation at 14 DPA, and reduced gains in bone regeneration at 21, 28, 35, and 42 DPA (***P = 0.000545; ***0.002952; ***0.002954; *0.049476 respectively) that are resolved by 63 DPA. (D) Representative μCT renderings of a single WT female (top) P3 digit and single Dp16 DS female (bottom) P3 digit prior to amputation and over the duration of the regeneration response. Blue arrowheads demonstrate similar onset of blastema osteogenic differentiation and mineralization. (E–F) Dp16 DS females show transiently attenuated regenerated bone length (**P = 0.005707) and volume (***P = 0.004869) at 21 DPA that is resolved by 28 DPA. (For interpretation of the references to color in this figure legend, the reader is referred to the web version of this article.)

Next, to investigate the impact of DS on intramembranous bone regeneration, P3 amputations were performed on 8-week-old male and female Dp16 DS, Ts65Dn DS, and respective WT controls, followed by longitudinal *in vivo* μCT. The P3 amputation plane [15,16,23,26,44,47] (dashed lines in Figs. 1A and 2A, D) removes approximately 30 % of the pre-amputation bone length (Fig. 2B, E) but minimal bone volume (Fig. 2C, F), and does not transect the marrow cavity. P3 amputation triggers rapid osteocyte death at the amputation plane [17] that is associated with the initiation of osteoclast recruitment and activity to initiate removal of skeletal wound debris and expel the distal necrotic bone fragment (Fig. 2A, yellow arrowheads). Following osteoclast recruitment and bone resorption and subsequent expulsion of the necrotic bone fragment, blastemal osteoblast recruitment and bone formation *via* intramembranous ossification is initiated (Fig. 2A, blue arrowheads) that restores the entire P3 bone element as the surrounding soft tissue is regenerating [24,26,52]. Final P3 bone regeneration is characterized by a restoration of pre-amputation P3 skeletal length and an overshoot in bone volume due to enhanced deposition of bone along the dorsal ventral axis [26].

μCT analysis demonstrated that Dp16 WT males (n = 20 digits) progress through the resorption phase, complete by 14 DPA, and subsequently enter the bone formation/P3 regeneration phase by 21 DPA, resulting in robust osteoblastic bone formation and restoration of P3 at 42 DPA (Fig. 2A–C). In Dp16 DS males (n = 12 digits), bone resorption was significantly enhanced, demonstrated by significantly reduced bone volume (but not bone length) at 14 DPA compared to WT males (Fig. 2C, t-test adjusted for multiple comparisons, 95 % CI). Dp16 DS males exited bone resorption and transitioned through the reversal phase (~14–21 DPA Fig. 2B) and into the bone formation/P3 regeneration phase by 21 DPA (Fig. 2B, C). However, Dp16 DS males had significantly reduced bone volume at all time points out to 42 DPA (Fig. 2C) and significantly reduced bone length at 21–42DPA (Fig. 2B) compared to Dp16 WT males. Intriguingly, the Dp16 DS male deficits in P3 bone regeneration evident at 42 DPA (Fig. 2B, C) had completely resolved by 63 DPA, with regenerated bone length and the overshoot in bone volume not significantly different between the two groups (Fig. 2C). Dp16 DS females (n = 8 digits) progressed through the bone resorption phase like Dp16 WT females with significantly decreased bone length and volume only at 21 DPA (Fig. 2E, F). This modest difference at 21 DPA was presumably associated with robust ossification in Dp16 WT females compared with ossification focused largely on the residual bone stump in Dp16 DS females (Fig. 2D, blue arrowheads). From 28 DPA to 63 DPA, the early differences in bone regeneration were resolved between both the Dp16 WT and DS females (Fig. 2E, F). These findings in Dp16 DS mice undergoing P3 regeneration are consistent with the decreased bone turnover we reported in Dp16 DS males at peak adult bone mass, that is absent in females [5].

**Fig. 3.**
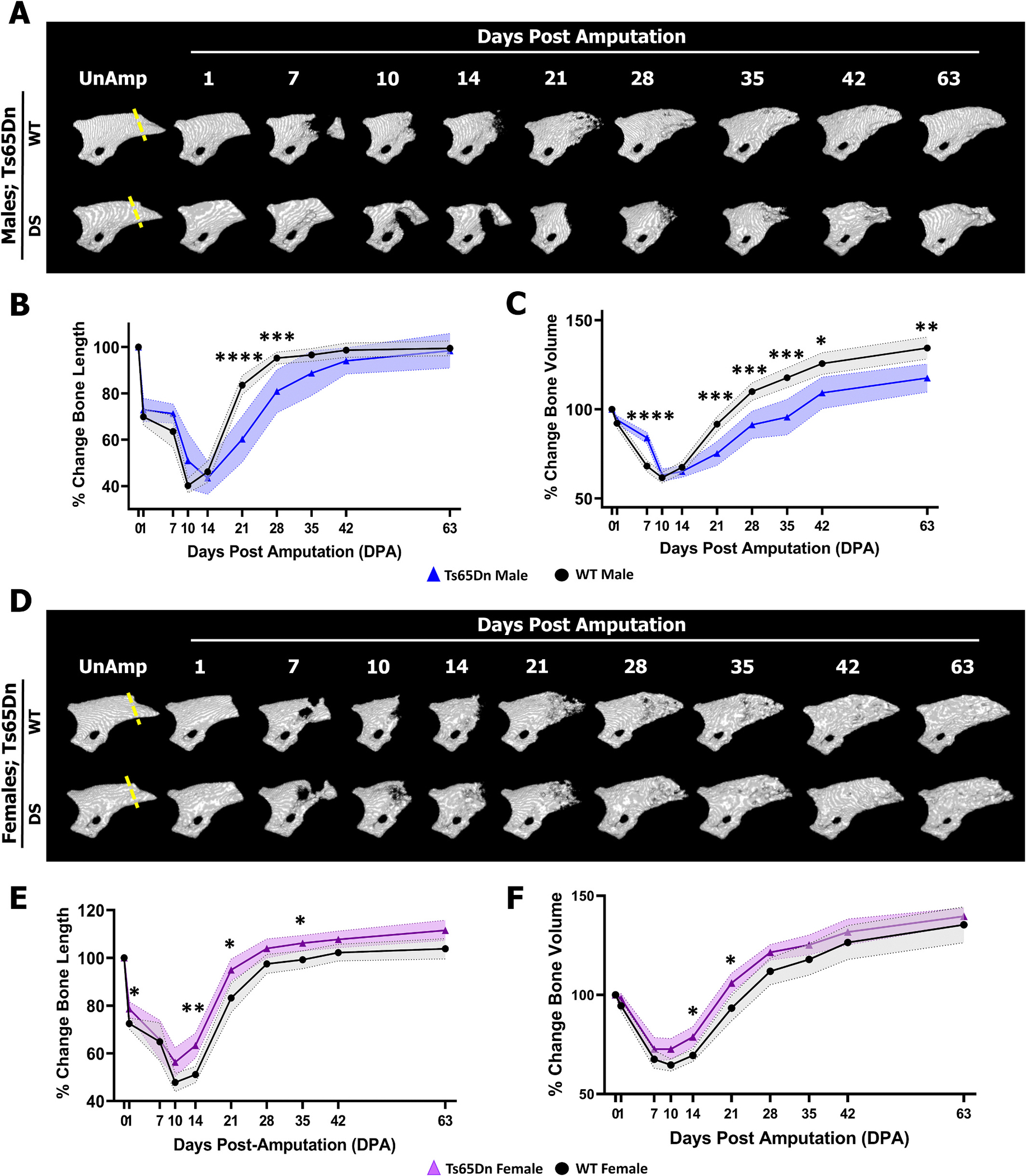
Sexually dimorphic P3 bone regeneration responses in Ts65Dn mice. Distal is to the right, dorsal is to the top. (A) Representative μCT renderings of a single WT male (top) P3 digit and a single Ts65Dn DS male (bottom) P3 digit prior to amputation (dashed line) and over the course of regeneration. P3 regeneration is attenuated in Ts65Dn DS males. (B) Ts65Dn DS male digits show reduced regenerating bone length at 21 (****P = 0.000065) and 28 DPA (***P = 0.001516). (C) P3 bone degradation is reduced in male Ts65Dn DS digits at 7 DPA (****P = 0.000004) and P3 bone regeneration is reduced at 21 (***P = 0.000551), 28 (***P = 0.000751), 35 (***P = 0.000751), 42 (*P = 0.010053), and 63 DPA (**P = 0.008763). (D) Representative μCT renderings of a single WT female (top) P3 digit and a single Ts65Dn DS female (bottom) P3 digit prior to amputation (dashed line) and over the course of regeneration. (E) P3 bone length is transiently increased at 14 (**P = 0.001958), 21 (*P = 0.019053) and 35 DPA (*P = 0.031643) in Ts65Dn DS females compared to WT females. (F) Ts65Dn DS female P3 bone volume is transiently enhanced at 14 (*P = 0.020120) and 21 DPA (*P = 0.018817) compared to WT females.

To investigate the genetic variability of P3 bone regeneration in the DS setting, P3 amputations were performed on 8-week-old male and female Ts65Dn DS mice and Ts65Dn WT controls and followed by weekly *in vivo* μCT analysis (Fig. 3). While both Ts65Dn WT (n = 16 digits) and Ts65Dn DS (n = 8 digits) males progressed through bone degradation at 10–14 DPA, the resorption phase was significantly and transiently attenuated in Ts65Dn DS males (Fig. 3A, C), resulting in less resorption of bone volume at 7 DPA compared to WT males. Like Dp16 DS males, P3 bone regeneration was markedly reduced in Ts65Dn DS males compared to Ts65Dn WT males, resulting in significantly decreased bone length at 21 and 28 DPA (Fig. 3B), and bone volume at 21, 28, 35, 42, and 63 DPA (Fig. 3C). However, unlike Dp16 DS mice, the bone regeneration deficits in Ts65Dn DS males did not resolve by 63 DPA. Instead, Ts65Dn DS males did eventually regenerate, as they fully restored the amputated P3 length (Fig. 3B), yet the expected overshoot in bone volume was significantly reduced compared to Ts65Dn WT males (Fig. 3C). Conversely, Ts65Dn females (n = 16 digits) progressed through the entire degradation phase indistinguishable from Ts65Dn WT females (n = 16 digits), albeit with some transiently enhanced regeneration of bone length and bone volume during the regeneration phase (Fig. 3D–F). Taken together, these findings demonstrate the dimorphic response in Ts65Dn DS animals extends to bone injury and regeneration. As shown, males have a relatively poor capacity for P3 regeneration, characterized by attenuated resorption and decreased woven bone formation and regeneration, whereas Ts65Dn DS females are fully capable of P3 regeneration indistinguishable from Ts65Dn WT females by 42 DPA.

### 3.2. Decreased osteoclast and osteoblast differentiation is associated with attenuated bone regeneration in Ts65Dn DS males

**Fig. 4.**
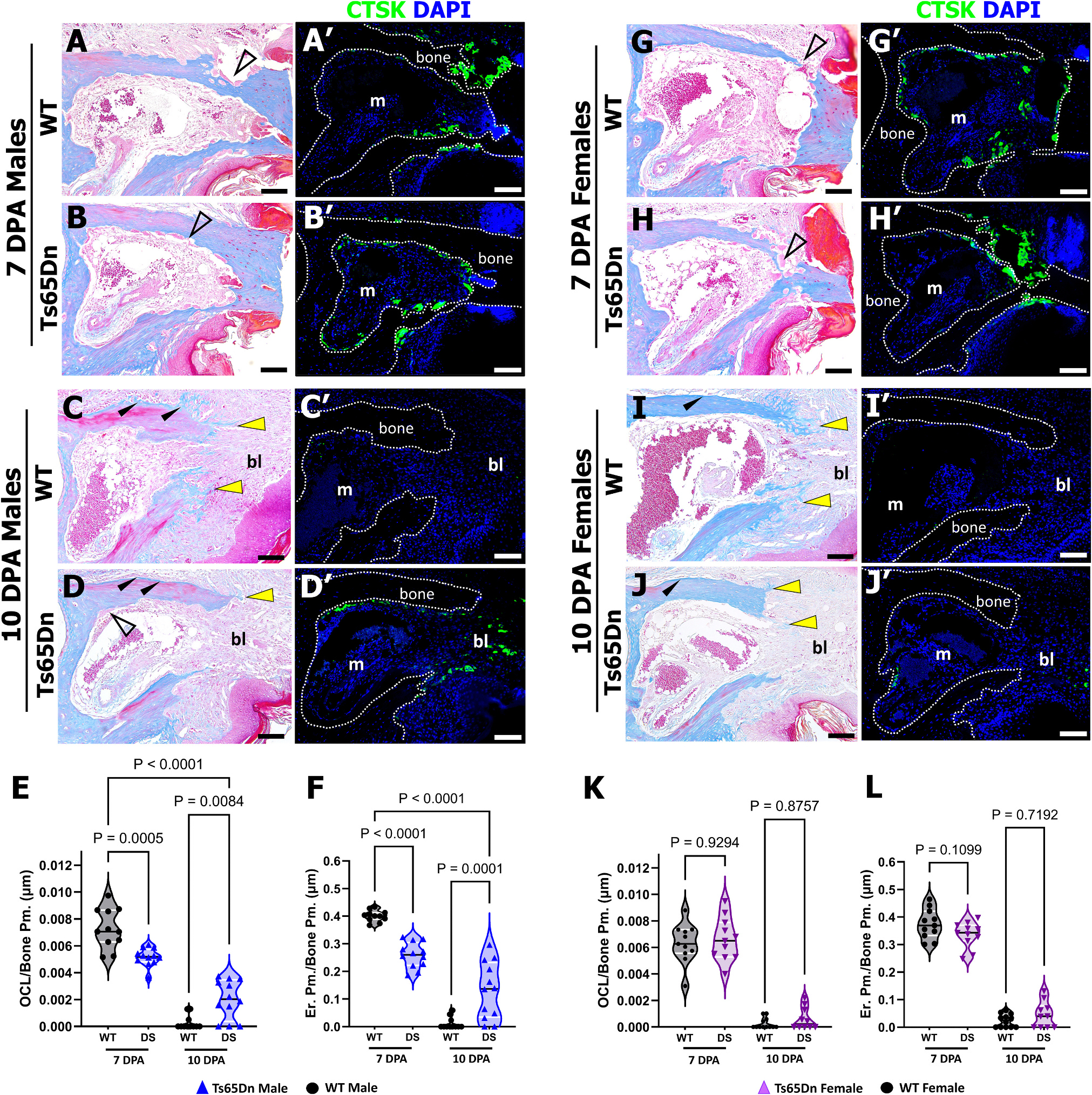
Sexually dimorphic osteoclast differentiation during P3 regeneration in Ts65Dn DS mice. Distal is to the right, dorsal is to the top. (A–A′) Serial sections of a representative 7 DPA WT male digit. (A) Mallory trichrome staining illustrating osteoclast erosion pits (open arrowhead) at 7 DPA. (A′) Cathepsin K (CTSK) immunostaining showing osteoclasts lining the degrading bone stump relative to the bone marrow (m) cavity. Scale bars = 200 μm. (B–B′) Serial sections of a representative 7 DPA Ts65Dn DS male digit. (B) Mallory Trichrome staining illustrating osteoclast erosion pits (open arrowhead at 7 DPA). (B′) CTSK immunostaining showing fewer osteoclasts lining the Ts65Dn DS male bone stump at 7 DPA. (C–C′) Serial sections of a representative 10 DPA WT male digit. (C) Mallory staining illustrating stump ossification (black arrowheads) and blastemal (bl) ossification (yellow arrowheads). (C′) CTSK immunostaining showing osteoclasts are absent at 10 DPA in WT male digits. (D–D′) Serial sections of a representative 10 DPA Ts65Dn DS male digit. (D) Mallory staining showing remaining osteoclasts (open arrowhead), attenuated stump ossification (black arrowheads), and the initiation of blastemal ossification (yellow arrowhead). (D′) CTSK immunostaining showing remaining osteoclasts in the 10 DPA Ts65Dn DS male digit. (E) Quantification of CTSK^+^ osteoclasts over P3 bone perimeter at 7 and 10 DPA demonstrates decreased osteoclast differentiation in Ts65Dn DS males. (F) Quantification of erosion perimeter over P3 bone perimeter illustrates decreased osteoclast activity in Ts65Dn DS males. Serial sections of a representative 7 DPA WT female digit (G–G′) and Ts65Dn DS female digit (H–H′). (G, H) Mallory trichrome staining illustrating osteoclast resorption pits (open arrowhead) at 7 DPA. (G′, H′) CTSK immunostaining showing osteoclasts lining the degrading bone stump. Serial sections of a representative 10 DPA WT female digit (I–I′) and Ts65Dn DS female digit (J–J′). (I, J) Mallory trichrome staining showing stump ossification (black arrowhead) and blastemal ossification (yellow arrowheads). (I′, J′) CTSK immunostaining showing osteoclasts are absent in 10 DPA females of WT and DS mice. Quantification of CTSK+ osteoclasts over P3 bone perimeter (K) and erosion perimeter over P3 bone perimeter (L) reveals no differences in osteoclast number or activity between WT and Ts65Dn DS females at either time point. (E, F, K, L) Violin plots showing individual data points, medians and 1st and 3rd quartiles resulting from two-way ANOVA, with Tukey’s multiple comparisons test. OCL, osteoclast number; Bone Pm, bone perimeter; Er Pm, erosion perimeter. Immunohistochemical stained samples counterstained with DAPI (blue). (For interpretation of the references to color in this figure legend, the reader is referred to the web version of this article.)

To interrogate the cellular drivers of the altered bone resorption response in male and female Ts65Dn DS mice, amputated digits from both male and female Ts65Dn DS and WT littermates were harvested at 7 and 10 DPA (Fig. 4). For reference, digits at 7 DPA correspond to the robust bone degradation stages [17,24] and early blastema formation [17], whereas digits at 10 DPA are associated with the resolution of bone degradation and the initiation of both stump re-ossification [17] and early blastema-driven bone formation [26]. At 7 DPA, histological and immunohistochemical analysis demonstrated significantly decreased Cathepsin K+ (CTSK) osteoclasts and decreased erosion surface (open arrowheads) in Ts65Dn DS males (n = 12 digits) compared to Ts65Dn WT males (n = 11 digits) (Fig. 4A–B′, E, F). By 10 DPA, the digits of both Ts65Dn DS and WT males showed a prominent blastema (yellow arrows), yet Ts65Dn WT males (n = 11 digits) showed markedly reduced CTSK+ cells and erosion surface and an associated increase in bone (stained blue) associated with the stump (black arrowheads) that extended into the blastema (yellow arrowheads) (Fig. 4C–F) compared to Ts65Dn DS males. Indeed, Ts65Dn DS males (n = 12 digits) showed significantly elevated osteoclasts and erosion surface (open arrowhead) compared to Ts65Dn WT males, but with regions of stump ossification (arrowheads) and blastemal ossification (yellow arrowhead) also apparent (Fig. 4C–F). To determine whether Ts65Dn males were temporally delayed in the capacity for osteoclast differentiation and activity *in vivo*, our analysis was extended and 7 DPA Ts65Dn WT males were compared with 10 DPA Ts65Dn DS males. This comparison identified that these time points were not equivalent, supporting our hypothesis of a decreased, and not simply delayed, resorptive phase in Ts65Dn DS male mice (Fig. 4E, F, Two-way ANOVA, with Tukey’s multiple comparisons test). WT osteoclast number and eroded surface were negligible, indicating completion of the resorption phase at 10 DPA, whereas Ts65Dn males had persistent osteoclast number and eroded surface at 10DPA. Conversely, WT and Ts65Dn DS females showed no differences in any of the measured osteoclast parameters (number of CTSK+ cells and erosion surface (open arrowheads)) at 7 DPA (Ts65Dn WT: n = 12 digits and Ts65Dn DS: n = 11 digits) or 10 DPA (Ts65Dn WT: n = 12 digits and Ts65Dn DS: n = 10 digits) (Fig. 4G–L). These P3 data are entirely consistent with our previous findings of reduced osteoclast number and eroded bone surface in the basal bone phenotype in the tibia and femur of Ts65Dn DS male mice [31]. Collectively, these findings support the conclusion that the altered P3 degradation response in Ts65Dn DS males is driven by inherent deficits in the capacity for osteoclast differentiation and activity *in vivo*, regardless of homeostatic or injury conditions.

**Fig. 5.**
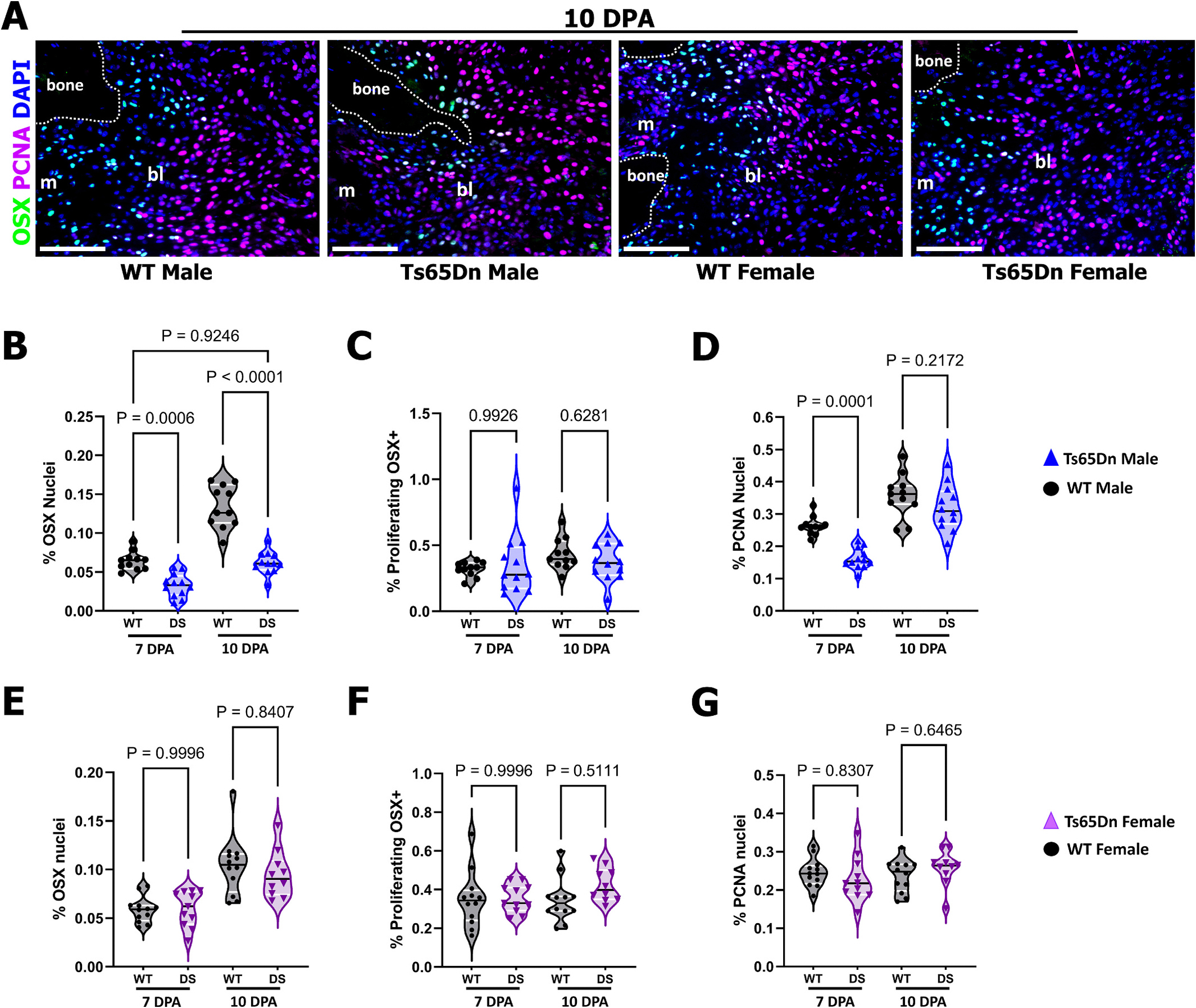
Attenuated osteogenesis in Ts65Dn DS male mice. (A) Representative 10 DPA digits co-immunostained for osterix (OSX) as an indicator of osteogenesis, proliferating cell nuclear antigen (PCNA), and DAPI to visualize nuclei in blue. Distal is to the right, dorsal is to the top. Scale bars = 200 μm. Osteogenesis is associated with the stump at 10 DPA and extends into the blastema (bl). Marrow (m). Scale bars = 50 μm. Quantification of OSX^+^ osteoblasts (B) reveal attenuated osteogenesis in Ts65Dn DS males compared to WT males at both 7 and 10DPA, whereas no differences were observed in proliferating PCNA^+^/OSX^+^ osteoblasts (C) and total proliferating cells (D). In females (E–G), no differences were observed in osteoblasts (E), proliferating osteoblasts (F) or total proliferating cells (G). Violin plots showing individual data points, medians and 1st and 3rd quartiles resulting from two-way ANOVA, with Tukey’s multiple comparisons test. (For interpretation of the references to color in this figure legend, the reader is referred to the web version of this article.)

Next, to examine woven bone formation and the osteogenic phase of P3 regeneration, co-immunostaining of P3 digits for the osteoblast marker, Osterix (OSX) and Proliferating Cell Nuclear Antigen (PCNA) in both male and female Ts65Dn DS and WT digits at 7 and 10 DPA (Fig. 5) was performed. We have previously shown that early osteogenesis is associated with the P3 stump and gradually extends into the blastema in a proximal-to-distal fashion to restore the P3 element [17,24,26,52]. At 10 DPA in Ts65Dn WT males, OSX^+^ cells were associated with both the bone stump and extended into the blastema, whereas Ts65Dn DS males had OSX^+^ cells primarily localized to the bone stump (Fig. 5A). Osteoblast quantification showed significantly reduced OSX^+^ cells at 7 and 10 DPA in Ts65Dn DS males (n = 12 digits) compared to Ts65Dn WT males (n = 11 digits). However, the proportion of proliferating osteoblasts (*i.e.*: OSX^+/^PCNA^+^ cells) was similar between Ts65Dn DS and WT males at both 7 DPA and 10 DPA (Fig. 5B). While no significant differences in osteoblast proliferation were observed, total PCNA^+^ cells were significantly decreased at 7 DPA in Ts65Dn DS male digits compared to Ts65Dn WT males (Fig. 5C). Interestingly, these differences in cell proliferation were fully resolved by 10 DPA (Fig. 5D). Comparison of the number of OSX^+^ cells at 7 DPA in WT males and at 10 DPA in Ts65Dn DS males suggested a delay in the initiation of osteogenic differentiation (Fig. 5B; Two-way ANOVA, with Tukey’s multiple comparisons test), where 10 DPA Ts65Dn DS mice were not different from the Ts65Dn WT mice at 7DPA. The μCT data (Fig. 3) support the idea that Ts65Dn DS males also have a reduced capacity for osteoblast differentiation, rather than merely delayed initiation of osteogenesis. InTs65Dn DS and WT females, no differences in OSX^+,^ PCNA^+^ or OSX^+^/PCNA^+^ cells were observed at 7 and 10 DPA (Fig. 5A, E–G). Collectively, these findings suggest that the attenuated P3 bone regeneration response in Ts65Dn DS males is driven by decreased osteoclast differentiation and activity as well as reduced osteoblast differentiation, similar to our previous findings in *ex vivo* Ts65Dn bone marrow cultures [31,32].

### 3.3. Delayed wound healing in Ts65Dn DS mice

**Fig. 6.**
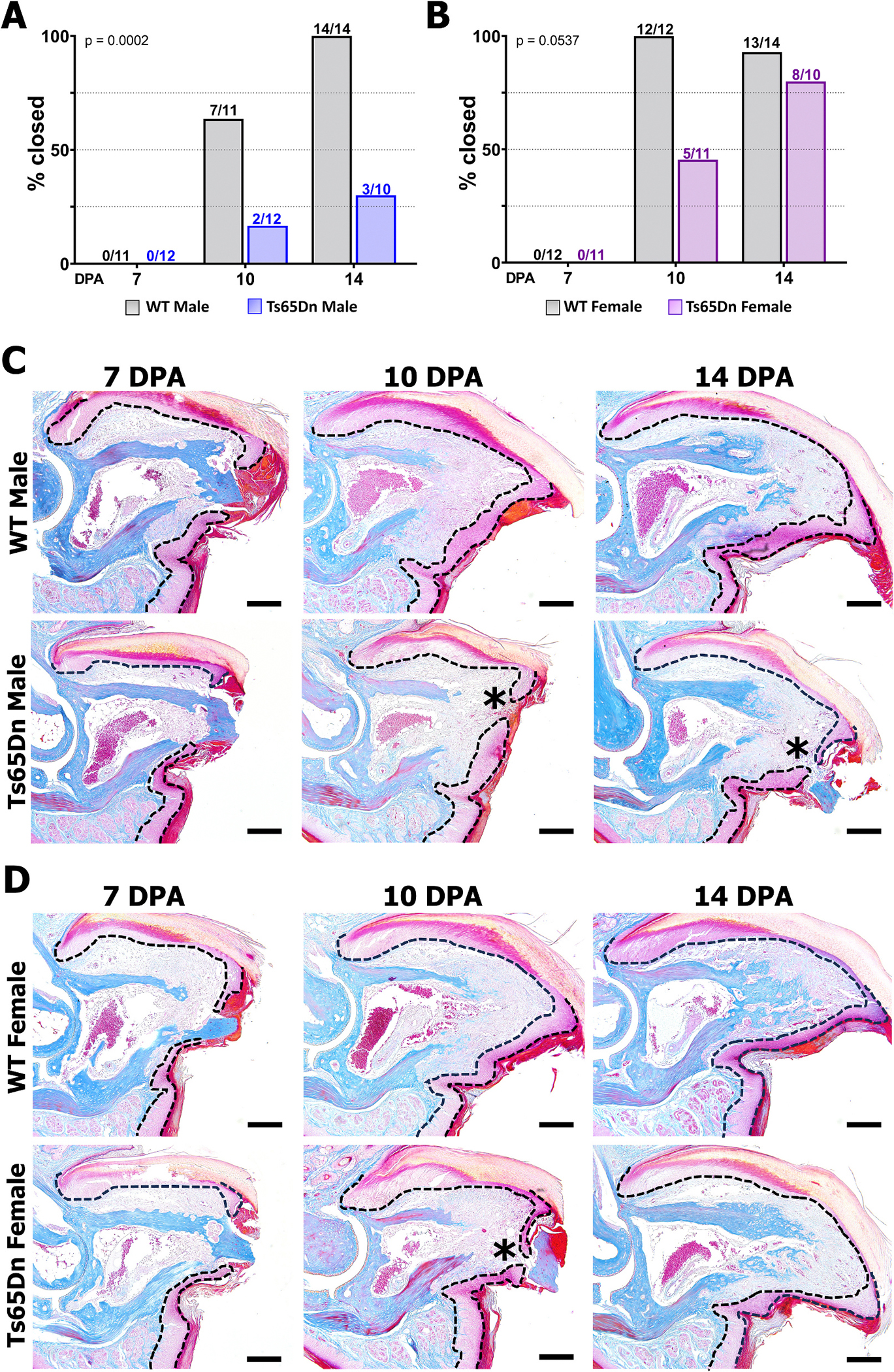
Delayed wound healing in Ts65Dn DS mice. (A-B) Percentage and number of digits with closed wounds at 7, 10 and 14 DPA in WT and Ts65Dn DS males (A) and females (B) (number of assessed wounds at each time point = 100 %). Graphs indicate percentage of digits with closed wounds, and values above bars indicate the number of closed digits over total number of digits assessed at each time point per group. P values indicate significance across all assessed time points. (C) Mallory Trichrome staining of representative WT and Ts65Dn DS male digits at 7, 10, and 14 DPA. Epidermis indicated by dashed line. Distal is to the right, dorsal is to the top. Scale bars = 200 μm. Asterisks at 10 and 14 DPA indicate delayed wound closure in Ts65Dn DS males, consistent with panel A. (D) Mallory Trichrome staining of representative WT and Ts65Dn DS female digits at 7, 10, and 14 DPA. Consistent with panel B, the asterisk at 10 DPA indicates delayed wound closure in Ts65Dn DS females.

Individuals with Ts21 have been suggested to have an increased incidence of wound healing complications, frequently associated with postoperative surgical complications [53]. However, direct experimental evidence of differences in wound healing phenotypes in DS are scarce in the literature, with some suggesting Ts21 pediatric patients have neither higher medical nor surgical complication rates after intestinal operations [54]. Since wound closure is a critical component of the P3 regenerative response [23,29] we next examined the wound healing response to the amputated P3 digit in male and female Ts65Dn WT and DS mice (Fig. 6).

Analysis of wound closure at 7, 10, and 14 DPA in Ts65Dn WT males demonstrated that wound closure was complete in 7/11 digits by 10 DPA and 14/14 digits by 14 DPA, whereas Ts65Dn DS males showed significantly delayed wound closure (by Fisher’s exact group comparison test, p = 0.0002) with closed wounds in 2/12 digits at 10 DPA and 3/10 digits by 14 DPA (Fig. 6A, C). Female Ts65Dn DS and WT mice ultimately regenerate the amputated P3 digit at the same capacity (Fig. 3D–F), yet wound closure was modestly attenuated (p = 0.0537) in Ts65Dn DS females (Fig. 6B, D). In Ts65Dn WT females, wound closure was complete by 10 DPA in all digits assayed and in 13/14 digits at 14 DPA. In contrast, Ts65Dn DS females exhibited delayed wound closure with only 5/11 healed wounds at 10 DPA and 8/10 at 14 DPA (Fig. 6B). Given that wound closure is similar during the initiation of regeneration phase (10 DPA) in Ts65Dn males and females, these data suggest that the reduced capacity for bone resorption and P3 regeneration in Ts65Dn males is not driven by delayed wound closure.

### 3.4. Biphasic P3 regeneration

**Fig. 7.**
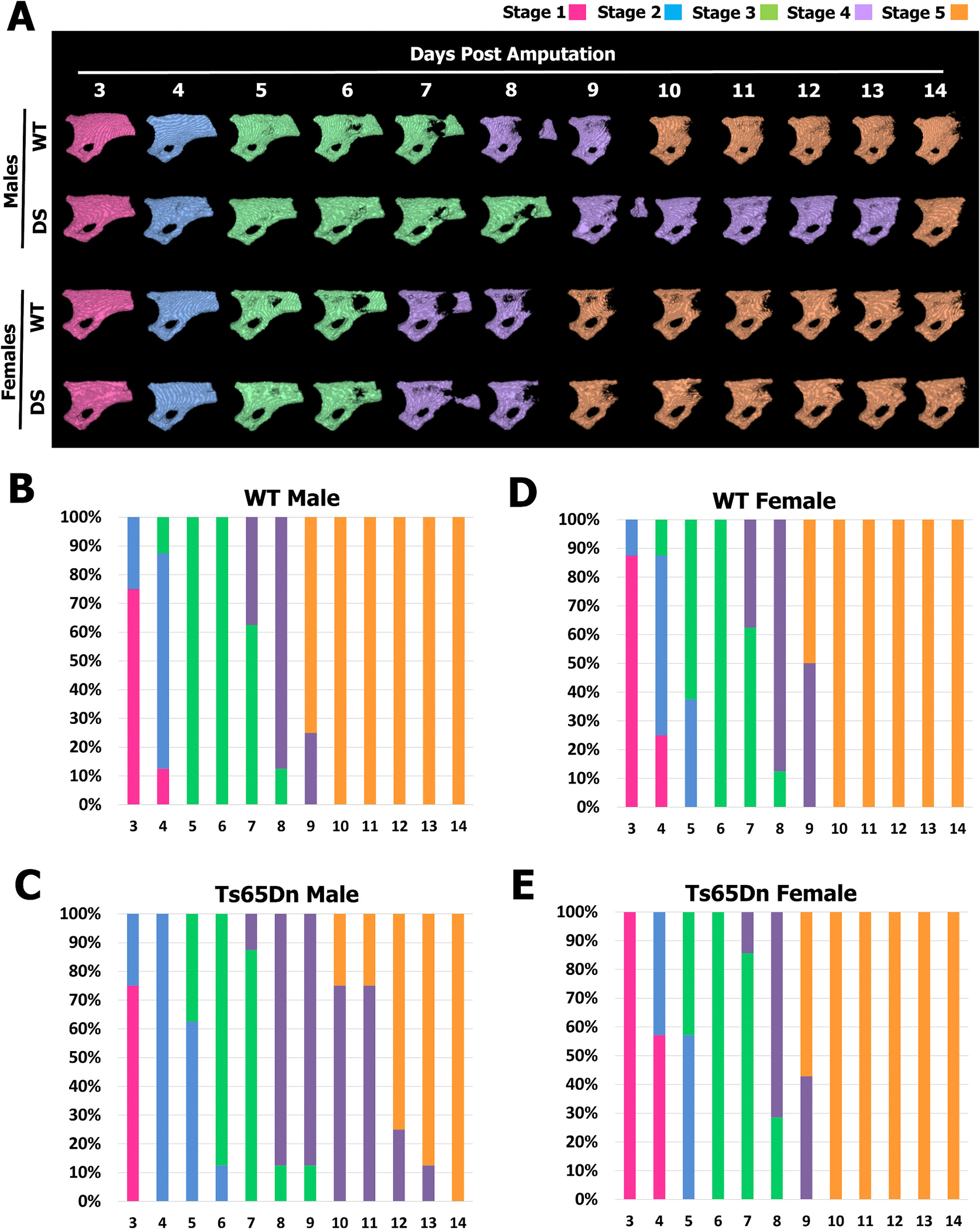
Digit staging showing altered osteoclast and osteoblast programs in Ts65Dn DS males. (A) Daily μCT renderings of regenerating digits from 3 to 14 DPA demonstrate progression to stage 2 (onset of bone pitting, blue) is indistinguishable between all genotypes, while Ts65Dn DS males are transiently delayed in stage 3 (robust bone degradation, green) and stalled in stage 4 (severing of the bone stump, purple) before initiating the final stage 5 of bone formation. Distal to the right, dorsal is to the top. (B–C) The rate of digit (n = 8) progression through stages 1–5 in WT (B) and Ts65Dn DS (C) male mice demonstrates the temporary delay in completion of stage 4. (D–E) The rate of digit (WT: n = 8, Ts65Dn DS: n = 8) progression through stages 1–5 are indistinguishable in females. (For interpretation of the references to color in this figure legend, the reader is referred to the web version of this article.)

Given the difference in progression through the regeneration response in Ts65Dn DS males, the P3 bone regeneration response was examined using a digit staging model we recently developed [17]. This model utilizes *in vivo* μCT imaging and captures 5 distinct stages of P3 regeneration that encapsulate the phenotypic progression of bone resorption and the initiation of bone regeneration regardless of the DPA [17]. Stage 1 is characterized by the smooth P3 bone surface, Stage 2 by osteoclast resorption pits identified along the bone surface, Stage 3 by osteoclast degradation through the cortical bone, Stage 4 by bone stump bifurcation, and Stage 5 by the initiation of bone islands [17] (Fig. 7A). Daily *in vivo* μCT imaging revealed that progression to Stage 2 was temporally indistinguishable between Ts65Dn DS and WT males, demonstrating that osteoclast recruitment and the initiation of bone resorption occurred within the same time frame, yet osteoclast resorption (Stage 3) was prolonged in Ts65Dn DS males (Fig. 7B, C). Stage 4 identifies osteoclast-mediated destruction of the remaining P3 bone stump. The osteoclast-mediated bifurcation process essentially creates a path for wound closure to proceed [24]. Ts65Dn DS male regeneration stalled briefly in Stage 4 (Fig. 7A, C), supporting earlier evidence that digit wound closure was significantly delayed in Ts65Dn DS males (Fig. 6A). In contrast, staging of P3 regeneration in Ts65Dn DS and WT females identified a virtually identical transition through all regeneration stages (Fig. 7D, E) highlighting the sexually dimorphic regeneration responses in male and female Ts65Dn DS mice.

## 4. Discussion

Trisomy 21 is one of the most complex genetic perturbations compatible with postnatal survival world-wide [55], occurring in approximately 1:700 live births [1]. In addition to being the leading cause of intellectual disability [56], the musculoskeletal impacts of Ts21 are diverse and highly variable, with many co-morbidities demonstrating variable prevalence relative to non-Ts21 people [2]. A recent retrospective assessment of ~2500 Ts21 individuals demonstrated increased fracture incidence in Ts21 individuals compared to ~12,000 euploid patients [14], that we had suggested previously [4,5,10,31]. Indeed, based on bone density measurements from Ts21 individuals, we, and others [4,57] speculated that low bone mass (that predisposes individuals to fracture risk) may also predispose to impaired bone healing. However, the literature is sparse regarding the mechanism driving the low bone mass seen in Ts21 and whether the inherent low bone mass predicts a decreased ability of bones to heal properly. To this end, Dp16 DS mice have significantly impaired fracture healing with enhanced inflammation potentially mediating a deficit in cartilage development and soft callus formation [5]. However, given the diminished bone formation rate and low bone mass that we and others have reported in Ts65Dn DS mice [5,31–33,39,50], we were interested to determine whether intramembranous bone formation would also be impaired in DS mice upon injury. Therefore, 8-week-old Dp16 and Ts65Dn DS male and female mice and WT controls were subjected to distal amputation of the terminal phalanx (P3) and the regeneration response assessed *via* longitudinal *in vivo* μCT imaging, histological, and immunohistochemical analysis. These data demonstrate that intramembranous bone regeneration is transiently attenuated in Dp16 DS male mice. In Ts65Dn DS males, both P3 bone resorption and intramembranous bone regeneration are decreased, similar to our previous findings in *ex vivo* Ts65Dn DS bone marrow cultures [31,32]. Importantly, impaired fracture healing in Dp16 DS mice is driven by reduced cartilaginous callus bridging of the fracture gap [5], while the current study provides evidence that osteoblasts are also perturbed in DS male mice. Thus, deficits are observed in both chondrocytes and osteoblasts in DS mice in response to injury. As such, these findings demonstrate that the diminished bone formation rate and low bone mass reported in these animals is consistent with a decreased capacity for the bones to heal properly post injury. Ongoing studies are focused on defining the cellular and molecular mechanisms that govern altered bone repair and regeneration in DS mouse models.

Sexual dimorphism is a common feature of many DS phenotypes, yet no clear explanations currently exist [12]. In Ts21 men with low testosterone, we have shown that the hypothalamic-pituitary-gonadal axis is indeed functional and that LH and FSH responses remain intact, though suppressed [5]. Ts21 individuals collectively present with a sexually dimorphic and variable spectrum of low BMD [4]. As an example, in Ts21, lumbar spine BMD is significantly decreased in males compared to females, and males have a significantly enhanced risk of developing low BMD at an earlier age than females [8]. This sexually dimorphic phenotype has led to speculation that the female skeleton presents with a relative “transient protection” against the more adverse Ts21-related skeletal phenotypes observed in Ts21 males [12], which is entirely consistent with our findings of sexually dimorphic P3 regeneration in Ts65Dn mice. In DS mice, sexual dimorphism has been reported previously regarding gene dosage and bone abnormalities in Dp1Tyb mice DS mice [58], low bone mass and volume in Dp16 DS males that is absent in Dp16 DS females [5], sexually dimorphic skeletal deficits in male and female Ts65Dn DS mice [38,50], and altered behavioral development and brain circuitry [59]. Our studies expand the growing database of sexual dimorphism in DS mice to include altered bone resorption and impaired direct bone formation following P3 amputation only in male Ts65Dn DS mice. The current study is the first *in vivo* evidence identifying sexually dimorphic outcomes to skeletal injury in Ts65Dn DS mice. Our ongoing studies are directed at identifying the cellular and molecular drivers of the sexually dimorphic bone regeneration responses in DS mice.

The observed diminished wound healing response in Ts65Dn DS mice that is independent of the impaired P3 regenerative response is intriguing. Indeed, we and others have reported the critical importance of wound healing for P3 regeneration [22,26,29,60]. There is abundant literature supporting the idea that wound healing is impaired in Ts21 individuals, frequently associated with perioperative [61] or postoperative surgical complications [53]. Further investigation suggests that the adverse surgical outcomes are primarily infection-related (pneumonia, sepsis, and urinary tract infection) consistent with previous studies suggesting Ts21 patients are at increased risk for cellular and humoral immune deficiency, poor cell chemotaxis and reduced specific antibody responses [62]. In the case of P3 regeneration, both male and female Ts65Dn mice demonstrated diminished wound healing at 10 DPA, yet only males presented with diminished resorption and bone regeneration. Thus, the delayed response in Ts65Dn DS male mice was distinct from the impaired wound healing (as both males and females exhibited impaired wound healing) and reflects an intrinsic difference in the recruitment, differentiation and function of osteoclasts and osteoblasts. This cellular deficit has been reported by us and others [31–34,39] yet the mechanism(s) responsible remain unclear.

The utility of the P3 regeneration model is that it serves as an *in vivo* system to temporally and spatially investigate targeted bone resorption followed by bone formation (*i.e.* bone remodeling [63,64] of the P3 stump that merges with blastemal-derived osteoblasts that undergo intramembranous ossification [17,28,29]). Thus, the impairment of bone resorption and subsequent bone formation during P3 regeneration contributes to our understanding of poor bone healing (and associated risk of fracture) in Ts21 individuals, as well low bone accrual in this population. Despite the efforts of many investigators [2,4,7,8,12], there is a paucity of research into the extent of osteoporosis and/or osteopenia in Ts21 individuals. However, the data that does exist suggests that Ts21 individuals are at an increased risk of reduced BMD and increased fracture risk [14] and potentially delayed or impaired fracture healing [13]. In our case, we have been studying the spectrum of DS mouse models for over 15 years and have demonstrated that low bone accrual, low BMD, impaired fracture healing, and now impaired bone resorption and intramembranous bone regeneration, is a consistent feature in DS mice [5,31]. A recent human retrospective cohort analysis identified a positive association between Ts21 individuals and subsequent fractures in women >50 years of age [14]. This interesting study identified a low osteoporosis diagnosis rate in Ts21 individuals and suggests that factors other than osteoporosis contribute to the elevated fracture risk in older Ts21 people. This finding is entirely consistent with our previous studies that have suggested that low bone mass in Ts21 is due to low bone accrual and not osteoporosis [4]. In the case of adults with Ts21 who sustain an osteoporosis fragility fracture, current clinical recommendations suggest evaluation for secondary causes of osteoporosis and screening for medications associated with adverse effects on bone health and incorporating a shared decision-making approach [11]. This information combined with decreased fracture repair and P3 woven bone formation in DS mice, suggest that any fractures are likely to have difficulty healing. This potential bone healing problem was highlighted by a recent case study involving a 9-month-old Ts21 infant girl with an atrophic nonunion of the right humeral diaphysis [13]. In sum, evidence is accumulating that strongly suggests an important bone repair deficit is evident in Ts21 people and murine DS models. Providing care for the increasing number of Ts21 adults can be challenging, especially considering the broad phenotypic variation in health and function [11]. Collectively, a continued focus on bone health in Ts21 individuals is needed, as are ongoing studies investigating bone repair and regeneration at the cellular and molecular level across the lifespan.

## Data Availability

The data that support the findings of this study and Down syndrome mouse models are commercially available from Jackson Labs or from the corresponding author upon reasonable request.
